# Editor's Letter-Box

**Published:** 1899-08-26

**Authors:** 


### Editor's Letter-Box.

[Our Correspondents are reminded that prolixity is a great bar to publication, and that brevity of style and conciseness of statement greatly facilitate early insertion.]

"A Nurse" writes: In a paragraph in last week's Hospital on the subject of the number of deaths from typhoid fever among the troops in India, it is suggested that this appears to be caught in "native drinking shops and other objectionable institutions rather than in the barracks." But I should like to ask, if this is so, how do you account for the very great number of deaths from the same disease that occur among young officers, who presumably do not frequent native drinking shops? To take one station alone—Poona—the number of deaths amongst the officers is terrible; yet other Europeans seem to escape.

⁂ The question is a very proper one. We believe that the official view is the one stated, but there is much in regard to the spread of typhoid fever in hot countries which is at present but ill understood. What is clear is that the water carriage of typhoid by no means covers the whole ground, and it is not improbable that the dry-earth system, together with the swarms of flies which seem to be its inevitable accompaniment, may have much to do with the endemicity of typhoid fever in certain quarters. The question, however, which is put by "A Nurse" cannot as yet be answered satisfactorily.—Ed. *T. H.*

